# Smartphone applications for thoracic surgeons

**DOI:** 10.1186/1749-8090-8-S1-O323

**Published:** 2013-09-11

**Authors:** N Cassanelli, G Magnanelli, C Benato, B Canneto, G Falezza, A Lonardoni, G Silvestre, F Calabrò

**Affiliations:** 1 Thoracic Surgery, AOUI, Verona, Italy

## Background

Rapid expansion of smartphones use in the clinical setting has been reported. Purpose of this review is to summarize the smartphone applications (apps) useful for thoracic surgeons.

## Methods

We’ve searched the online stores of the most popular smartphone (iOS, Android, Blackberry, Windows) for specific thoracic surgery apps. Search terms were: thoracic surgery, lung cancer, esophageal, chest, surgery; data were then stored and analyzed.

## Results

We’ve found a total of 159 apps; we’ve excluded from our study 85 apps, related to patient education and basic medical knowledge, and 22 apps designed for students. Fifty-two apps were elegible for our study, all of them reporting a medical involvement or a bibliographic support. The topics were online version of journals (10 apps), TNM staging tools (6), risk assessment (5), radiology tools (9), pulmonary function test evaluation (3), past cardiothoracic annual meetings (6), guidelines/online resources (9), self-evaluation quiz (4). Three apps (6%) were released in 2010, 9 (17%) in 2011, 28 (54%) in 2012 and 12 (23%) in 2013. Forty-six apps (88%) were free of charge. Six (12%) had a price ranging from 0.89 to 17.99 € (average 5.39 €). Only three (6%) had customer satisfaction rating (5-16 rates), with an average vote of 4.3 out of 5 (range 3-5).

## Conclusion

Smartphone apps can improve education, diagnostic and therapeutic skills of thoracic surgeons. Initial data from this study can be useful for thoracic surgeons to try and rate smartphone apps in their daily practice, guiding developers to release updated and high-quality apps.

